# A multicentric real-world observational study to describe the use and efficacy of follitropin delta for IVF/ICSI procedures in patients at risk of hypo-response

**DOI:** 10.3389/frph.2025.1650946

**Published:** 2025-09-10

**Authors:** Anne-Claire Deloire, Géraldine Porcu-Buisson, Romane Lefebvre, Mathilde Bernot

**Affiliations:** ^1^ Clinic of Diaconat-Roosevelt, Colmar, France; ^2^ Department of Reproductive Medicine, Institut de Médecine de la Reproduction, Marseille, France; ^3^ Ferring S.A.S., Gentilly, France

**Keywords:** follitropin delta, ovarian hypo-response, real-word evidence, pregnancy, ovarian stimulation

## Abstract

**Background:**

Around 20% of patients undergoing assisted reproductive technology are at risk of hypo-response to ovarian stimulation. The aim of this study was to describe the real-world use of follitropin delta for ovarian stimulation in these patients, as defined by POSEIDON groups 3 and 4 [an anti-Müllerian hormone (AMH) level of <1.2 ng/ml].

**Materials and methods:**

This study was a *post-hoc* analysis of participants from DELTA, a multi-centre, prospective, observational study conducted in normal care settings in fertility clinics at 14 active sites in France. A subset of 35 patients at risk of hypo-response to ovarian stimulation (mean AMH 0.7 ± 0.29 ng/ml) and treated with follitropin delta were included. Patients were followed for 10–11 weeks after the first fresh or frozen embryo transfer in case of subsequent pregnancy, and data on real-world follitropin delta use collected.

**Results:**

Most patients (92.9%) had undergone their first IVF or ICSI. The prescribed daily dose was usually based on the approved algorithm (*N* = 26; 74.3%) with a mean daily dose of 14.2 ± 4.1 mcg, resulting in a mean total dose of 187.7 ± 135.6 mcg. The mean duration of ovarian stimulation was 11.6 ± 6.7 days with no premature discontinuations, while the mean number of oocytes retrieved among patients that started stimulation was 6.3 ± 4.3. A fresh transfer was performed for 21 patients (84.0%), with a mean of 1.04 ± 0.98 embryos transferred per patient. Seven patients (20.0%) achieved an ongoing pregnancy (28% per transfer). No adverse drug reactions were reported.

**Conclusions:**

The results describe the real-world use of follitropin delta and demonstrate its suitability for POSEIDON group 3 and 4 patients. These data complement clinical trial outcomes, supporting clinician decision-making and improving IVF/ICSI outcomes.

## Introduction

It is estimated that 1.9% of child-seeking women aged 20–44 years suffer from primary infertility (unable to have a live birth) and 10.5% secondary infertility (unable to have a second child or more) (1). Worldwide, one couple in six is believed to be affected by infertility (2). Couples that have difficulties conceiving may turn to assisted reproductive technologies (ART) such as *in vitro* fertilization (IVF) and intracytoplasmic sperm injection (ICSI) to achieve pregnancy. These techniques involve controlled ovarian stimulation (COS) by injection of gonadotropins to obtain an adequate number of competent oocytes to be used for the procedure at minimum risk to the patient (3).

Follitropin delta is the most recent gonadotropin to receive marketing authorization approval in European countries. It is the only gonadotropin on the market that is a human cell line-derived recombinant follicle stimulating hormone (FSH) and, to optimize efficacy and safety, uses a recommended dosing algorithm based on the patient's weight and ovarian response, as estimated by serum anti-Müllerian hormone (AMH) level. This individualized dosing is necessary to minimize the risk of ovarian hyperstimulation syndrome (OHSS) without compromising the chances of a successful pregnancy. Dosed using this algorithm, follitropin delta was found to be non-inferior to conventionally-dosed follitropin alfa with regards to ongoing pregnancy (4, 5) and to conventionally-dosed follitropin beta with regards to number of oocytes retrieved (6). Additionally, more women achieved the target ovarian response of 8–14 oocytes and had less need for OHSS preventive measures after treatment with follitropin delta (4–6). Further, in the DELTA real-world observational study, follitropin delta was usually administered based on the recommended algorithm and efficacy and safety were consistent with the results of phase III studies (7).

Patients with a low response to ovarian stimulation are at greater risk of failure in ART, with a cumulative live birth rate of only 7% based on the Bologna classification (8). Also, it has been demonstrated that, on average, the success rate in poor responders to ovarian stimulation is around 50% lower than in non-poor responders (9). One promising finding from DELTA study was that poor responders to ovarian stimulation, as defined by serum levels of AMH (< 1 ng/ml, *n* = 31), achieved an average ovarian response of 6 oocytes and an ongoing pregnancy rate per transfer of 31.8% (7). A more detailed analysis of treatment response in these individuals was thus warranted.

The POSEIDON classification has recently been introduced to better define ovarian response. POSEIDON categorizes patients into 4 groups based on clinical and biochemical parameters, to better streamline patient management. In particular, POSEIDON group 3 and 4 patients are defined as those with poor ovarian reserve pre-stimulation parameters (AFC < 5, AMH < 1.2 ng/ml) that are, respectively, younger than 35 years old or 35 years and older (10). The objective of this *post-hoc* analysis of the DELTA study was to describe the ovarian stimulation protocol used for POSEIDON group 3 and 4 patients and to characterize the efficacy and safety of follitropin delta for these groups. In view of previous encouraging results in these profiles with an unfavorable prognosis, a detailed analysis of the treatment protocol received by these patients and the intermediate efficacy and safety results could provide exploratory data that inform clinical practice and potentially improve outcomes in these difficult profiles.

## Materials and methods

### Study design

The present study was a *post-hoc* analysis of DELTA, a French, multicenter, prospective, observational study conducted between June 2020 and June 2021. The study was performed in compliance with the Declaration of Helsinki and current regulations, including local institutional review board ethics approval. Patients undergoing IVF/ISCI were treated according to routine clinical practice and enrolled after the decision to treat with follitropin delta had been made. No aspect of this study interfered with the routine medical procedures and/or medications received. The physician collected the non-objection to data collection in each patient’s medical record. The ClinicalTrials.gov identifier is NCT0450370.

### Study population

DELTA collected data from 248 patients undergoing their first or second attempt at IVF or ICSI and treated with follitropin delta for the first time. Women ≥18 years, who were either treatment-naïve or had undergone one previous IVF/ICSI treatment cycle and were prescribed follitropin delta for the first time, were consecutively included. Patients participating in an interventional clinical trial, requiring mandatory treatment or follow-up, or with a contraindication for follitropin delta treatment were excluded, as well as oocyte donors or women undergoing ovarian stimulation for fertility preservation.

For this *post-hoc* analysis, 47 patients with AMH levels <1.2 ng/ml in the DELTA study were first identified. Patients who did not meet the selection criteria or had no start date of treatment (*N* = 5) were excluded resulting in an analyzable population of 42. Of this population, patients that had not completed the treatment regimen or had received previous treatment with follitropin alpha (*N* = 7) were also excluded, leaving a per-protocol population of 35 patients at risk of hypo-response to ovarian stimulation (Supplementary Figure 1).

### Data collection

The investigators collected data for one stimulation cycle with follitropin delta. Women were followed-up until ongoing pregnancy (10–11 weeks) after the first fresh or frozen transfer. Baseline data included socio-demographic data: age, body weight, height, reproductive history, reasons for infertility, antral follicle count, most recent AMH test result, and laboratory measurements: FSH, luteinizing hormone (LH), estradiol and progesterone, as available.

Data collected at follow-up visits were the treatment protocol used, preventive measures for OHSS, ovarian response, number of oocytes retrieved and fertilized, number of embryos transferred, pregnancy outcomes: positive βhCG, clinical pregnancy (defined as the presence of at least one gestational sac 5–6 weeks after transfer) and ongoing pregnancy (defined as the presence of at least one intrauterine viable fetus 10–11 weeks after transfer), pregnancy loss, whether cycle cancelled and reason for cancellation, and serious and non-serious adverse drug reactions (SADRs and ADRs).

The study was performed in compliance with the Declaration of Helsinki, and current regulations. The study was submitted to the Committee for Personal Protection and received a favorable opinion. The physician collected the non-objection to data collection in each patient's medical record.

### Statistical analysis

Continuous/quantitative variables were described using the number of patients with data to be summarized (*n*), mean ± standard deviation (SD), median and interquartile range (IQR), and the number of missing data. Categorical variables were presented using frequency counts and percentage of each response. The total number of patients without missing data (*N* = 35) was used as the denominator for percentage calculations, unless otherwise specified. Only frequency counts were displayed for missing values. 95% CIs for means and percentages were provided, if relevant.

## Results

### Baseline patient characteristics

The per-protocol population included 35 patients at risk of ovarian hypo-response from 10 sites across metropolitan France. Most patients (*N* = 27, 92.9%) had undergone IVF or ICSI for the first time. The patient population presented a mean age of 35.6 ± 4.4 years, a body weight of 64.5 ± 8.3 kg, and a BMI of 23.7 ± 3.1 kg/m^2^. The mean duration of infertility was 3.5 ± 2.4 years. Most patients had primary infertility (*N* = 23, 65.7%). The mean AMH level was 0.7 ± 0.29 ng/ml (Table 1).

**Table 1:** Baseline characteristics of patients.

Characteristic	Post-hoc analysis (*N* = 35)
Age (years)	
All patients	35.7 ± 3.5
<35	10 (28.6)
≥35	25 (71.4)
Body weight (kg)	
All patients	64.5 ± 8.3
[50–60[	9 (25.7)
[60–70[	16 (45.7)
[70–80[	7 (20.0)
>80	3 (8.6)
BMI (kg/m²)	23.6 ± 3.0
Type of infertility	
Primary	23 (65.7)
Secondary	12 (34.3)
Duration of infertility (years)	3.5 ± 2.4
Reason for infertility^a^	
Anovulatory Infertility WHO Group I	1 (2.9)
Anovulatory Infertility WHO Group II	6 (17.1)
Tubal infertility	8 (22.9)
Uterine abnormality	1 (2.9)
Endometriosis	6 (17.1)
Unexplained infertility	7 (20.0)
Partner infertility	12 (34.3)
Other	8 (22.9)
Antral follicle count	10.4 ± 7.3
AMH (ng/ml)	0.70 ± 0.29
Follicle-stimulating hormone (IU/L)	8.04 ± 3.01
Luteinizing hormone (IU/L)	4.72 ± 1.85
Progesterone (nmol/L)	0.96 ± 1.09
Estradiol (pg/ml)	74.2 ± 48.7

Values are mean ± SD, median (interquartile range) or number (percentage), unless stated otherwise.

^a^ Patients could present more than one reason for infertility.

### Stimulation protocol and follitropin delta dosing

Of the 35 patients included, 32 (91.4%) were undergoing their first IVF/ICSI cycle (treatment-naïve) and 3 (8.6%) were undergoing their second IVF/ICSI cycle (non-naïve). The recommended dosing algorithm was used to calculate the starting dose in 26 patients (74.3%) and 24 (92.3%) patients maintained this dose during the stimulation period. The calculation tools (dosing app or website) were used for 22 of these patients (84.6%), and the calculated starting dose was modified prior to the first dose for only 2 (7.7%). The daily dose of follitropin delta of a further 5 patients (17.9%) was adjusted during the stimulation period. This was usually to target a higher number of follicles.

The mean daily starting dose of follitropin delta was 14.2 ± 4.0 mcg, and the mean total dose of follitropin delta administered 187.7 ± 135.3 mcg over a mean duration of 11.6 ± 6.7 days. Seven patients (20.0%) underwent a mixed protocol, receiving a mean dose of 129.7 ± 32.8 mcg of follitropin delta and 2,560.7 ± 923.1 IU of additional gonadotropins. Of these, a GnRH antagonist protocol was used in 80.0% of cases (Table 2).

**Table 2:** Stimulation protocol and follitropin delta use.

Characteristic	Post-hoc analysis (*N* = 35)
Previous IVF or ICSI for the current parental project	
No	32 (91.4)
Yes	3 (8.6)
Type of GnRH protocol used	
Antagonist	28 (80.0)
Agonist (long)	6 (17.1)
Agonist (short)	1 (2.9)
Mixed protocol	7 (20.0)
Dose based on the recommended algorithm	26 (74.3)
Starting daily dose maintained or modified compared to the recommended algorithm^a^	
Maintained	24 (92.3)
Increased	2 (7.7)
Decreased	0 (17)
Reason for initial dose modification^a^	
Targeting a higher number of follicles	4 (75.0)
Based on currently available additional parameters	1 (16.7)
Use of Dosing App or Website	22 (84.6)
Starting dose of follitropin delta prescribed (mcg)	14.2 ± 4.0
Mean daily dose of follitropin delta (mcg)	
Duration of ovarian stimulation with follitropin delta (days)	11.6 ± 6.7
Total dose of follitropin delta administered (mcg)	187.7 ± 135.6
Dose adjusted during ovarian stimulation	5 (14.3)
Reasons for dose adjustments^a^	
Ovarian hypo-response	4 (80.0)
Other	1 (20.0)
Cycle cancellation before oocyte pickup	3 (8.6)

Values are mean ± SD or number (percentage), unless stated otherwise.

^a^ Percentages for subgroups are based on subgroup totals.

### Oocyte retrieval and embryo transfer

Among the overall study population, a total of 32 patients (91.4%) had an oocyte pick-up with a mean number of 6.3 ± 4.3 oocytes retrieved. Among patients who underwent oocyte retrieval, 28 followed the algorithm, with a mean of 6.54 ± 4.26 oocytes collected, compared to 4.75 ± 5.19 in the 4 patients who did not. The target response (8–14 oocytes) was observed in 9 patients (28.1%), while 22 (68.7%) had an ovarian response below the target (< 8 oocytes). The response of one patient (3.1%) was above target (≥ 15 oocytes), with 20 oocytes retrieved. Overall, 28 patients (80.0%) obtained an ovarian response of 4–19 oocytes (Table 3).

**Table 3:** Oocyte retrieval, embryology, and pregnancy outcomes.

Characteristic	Post-hoc analysis (*N* = 35)
Patients with an oocyte retrieval	32 (91.4)
Total number of oocytes retrieved	6.3 ± 4.3
Ovarian response by category^a^	
<4 oocytes	9 (28.1)
4–7 oocytes	13 (40.6)
8–14 oocytes	9 (28.1)
15–19 oocytes	0
≥20 oocytes	1 (3.1)
Number of oocytes fertilized	3.8 ± 3.1
Number of D2-D3 embryos	3.2 ± 2.8
Number of D5-D6 blastocysts	1.0 ± 1.6
Patients with embryo transfer	25 (71.4)
Type of transfer^a^	
Fresh transfer	21 (84.0)
Frozen transfer	4 (16.0)
Number of embryos transferred^a^	1.04 ± 0.98
Patients with cancellation before oocyte pickup	3 (8.6)
Patients with cancellation after oocyte pickup	5 (14.3)
Implantation rate, %^b^**^,^**^c^	24.0
Positive βhCG per initiated cycle	10 (28.6)
Clinical pregnancy per initiated cycle	8 (22.9)
Ongoing pregnancy	7 (20.0%)
Pregnancy loss	1 (2.9)

Values are mean ± SD or number (percentage), unless stated otherwise.

^a^ Calculated based on the number of patients with embryo transfer.

^b^ Percentages for subgroups are based on subgroup totals.

^c^ Defined as the number of intrauterine viable fetuses after transfer divided by the number of embryos transferred.

A total of 25 patients (71.4%) received an embryo transfer, with a mean of 1.04 ± 0.98 embryos transferred. A fresh embryo transfer was performed in 21 patients (84.0%) while a frozen transfer was performed for 4 patients after a freeze-all strategy.

Three cycles (8.6%) were cancelled before oocyte pickup, all due to poor ovarian response. A further 5 cancellations (14.3%) occurred after oocyte pickup, due to no oocytes collected (*N* = 3), no oocytes fertilized (*N* = 2), and other medical reasons (*N* = 2).

### Pregnancy rate

Per started cycle, 10 patients (28.6%) tested positive for βhCG and clinical pregnancy (as assessed according to local practice) was reported for 8 patients (22.9%). The rate of clinical pregnancy per transfer was 28.0%. One patient (2.9%) reported pregnancy loss (Table 3).

Ongoing pregnancy was achieved in 7 patients (20.0%), all of whom were in the group that followed the algorithm. The ongoing pregnancy rate per embryo transfer according to age, mono or combined therapy, first or second initiated IVF/ISCI cycle and the transfer type are shown in Table 4.

**Table 4:** Ongoing pregnancy rates overall and by subgroup.

Characteristic	Post-hoc analysis (*n* = 35)
Ongoing pregnancy overall	7 (20.0)
By age (years)^a^	
<35	3 (30.0)
≥35	4 (16.0)
By mono or combined therapy^a^	
Follitropin delta only	5 (17.9)
Mixed protocol	2 (28.6)
By initiated IVF/ISCI cycle^a^	
First cycle (treatment-naïve)	6 (18.8)
Second cycle (non-treatment-naïve)	1 (33.3)
By transfer type^a^	
Fresh	6 (28.6)
Frozen	1 (25.0)

^a^ Percentages are calculated according to subgroup totals.

### Safety outcomes

No adverse drug reactions were reported.

## Discussion

The present study aimed to describe the use, efficacy and safety of follitropin delta after one cycle of treatment in patients at risk of hypo-response to ovarian stimulation undergoing IVF/ICSI in a real-world setting. Importantly, the study shows how follitropin delta is prescribed, used and followed for these subgroups. In addition, follitropin delta showed promising effectiveness without report of any adverse drug reactions.

Risk of ovarian hypo-response was defined for the purposes of this study as POSEIDON groups 3 and 4, for which AMH levels have been measured at less than 1.2 ng/ml. This subgroup of patients presented a mean AMH of 0.7 ng/ml, compared to a mean of 2.6 ng/ml in the DELTA study overall. Other baseline characteristics were similar, for example mean age, BMI, body weight and duration of infertility, as well as the proportions of patients with primary and secondary infertility. Although patients were not required to be undergoing IVF/ISCI for the first time, this was usually the case, as in the DELTA study overall. In turn, these characteristics were also similar to those of other real-world studies assessing the efficacy and safety of follitropin delta (11, 12).

Most patients in our study presented a primary infertility (65.7%), with a mean duration of 3.5 years at enrollment. In the DELTA study overall that was not restricted by ovarian response, the patient's partner was one of the reasons for infertility in nearly half of cases, similar to the findings of previous clinical trials (5, 12). As would be expected, this proportion was lower in the *post-hoc* study population (34.3%), where the partner was less likely to be the reason for infertility.

Regarding follitropin delta usage, around three-quarters of patients (74.3%) were prescribed a starting dose based on the recommended algorithm as compared to 88.3% in DELTA overall. Although lower in the present study, physicians did not usually consider the recommended dose to be insufficient for patients at risk of hypo-response. The mean starting dose of follitropin delta was higher among these patients than in DELTA overall (14.4 ± 5.2 mcg vs. 11.4 ± 4.1 mcg). This was above the maximum recommended daily dose of 12 mcg and may reflect the fact that dosing via the recommended algorithm was considered too cautious to achieve the target response. Accordingly, the total dose of follitropin delta administered was somewhat higher in the present study, 187.7 ± 135.6 mcg as compared to the 122.2 ± 80.0 mcg of the DELTA study overall and 90.0 ± 25.3 mcg in the ESTHER-1 trial, in which shorter durations of stimulations were observed (5). Importantly, the starting dose was modified for only 2 patients during the treatment regimen. Since most dose modifications in the DELTA study overall were reductions in response to the risk of ovarian hyperstimulation syndrome (OHSS), it follows that fewer would be observed among patients at risk of hypo-response, especially since the adjustment of gonadotropin dose is not recommended according to ESHRE guidelines (13).

Among the participants analyzed in the present study, the mean number of oocytes retrieved from those that started stimulation was 6.3 ± 4.3, and the target ovarian response (8–14 oocytes retrieved) was achieved by 28.1%. This was lower than the mean of 11.3 ± 6.8 oocytes retrieved and 46.1% target response in the DELTA study overall but closer to the 35.7% reported by Kovacs et al. (14), another real-world study of ovarian stimulation by follitropin delta in POSEIDON group 3 and 4 patients. The clinical pregnancy rate per cycle (22.9%) compared favorably to those of 21.6% for patients in group 3 and 16.7% in group 4 recently reported in a retrospective study of women undergoing treatment with ART (15). Ongoing pregnancy per started cycle, defined as the presence of one viable intrauterine viable fetus at 10–11 weeks), was achieved in 7 patients (20.0%). This was lower than in DELTA overall (29.6%) and in the PROFILE study (27.0%) (12) although these comparisons should be treated with caution due to the low number of POSEIDON group 3 and 4 patients available in DELTA. The fact that all ongoing pregnancies occurred in the group that followed the algorithm may be explained by the lower ovarian reserve and poorer prognosis of patients who did not follow it. No ADRs or SADRs were reported in this study. This is explained by the fact that most ADRs in the DELTA study overall were instances of OHSS, occurring particularly in patients undergoing a GnRH antagonist protocol (16).

This is the first real-world *post-hoc* analysis of follitropin delta for ovarian stimulation in patients at risk of ovarian hypo-response. The main strength of the study is the real-world prospective design, and therefore the representativeness of real clinical practice. For example, patients that had undergone one previous cycle of IVF or ICSI and treated with a different gonadotropin were included alongside treatment-naïve patients. Patients originated from 10 of the 14 centers included in the full DELTA study and no more than 6 originated from any one center. Also, baseline characteristics were as variable and as representative of the average patient population as in the DELTA study overall. Prospective studies such as this with primary data collection are less susceptible to missing data than some previous studies of ART that used a retrospective review of electronic medical records, with as many as 50% missing values for important clinical variables.

Certain limitations should also be considered. Firstly, the relatively high drop-out and cycle cancellation rates observed in this study may limit the strength of the conclusions and introduce potential bias. However, these rates likely reflect the specific characteristics of the patient population included in the trial, which may have contributed to greater treatment discontinuation. only a per-protocol subgroup of 35 patients at risk of ovarian hypo-response were able to be included from the broader DELTA population, which reflects the specificity of the target population (POSEIDON groups 3 and 4) and the *post-hoc* nature of the analysis. This limited number of patients inevitably reduces the statistical power and the ability to detect smaller differences or rare outcomes. It also restricts the generalizability of the findings to the broader population of hypo-responders. Nevertheless, the data presented here provide valuable real-world insights into the use of follitropin delta in a population where evidence is still limited. Where subgroups are reported, caution should be exercised in the interpretation of results. Limitations also relate to the real-world design, for example a lack of control of unmeasured variables, such as lifestyle factors or other pathologies. Finally, the results relate to the French metropolitan area only and may not be representative of other geographical regions.

## Conclusion

The results of the present study suggest that treatment with follitropin delta is suitable even for poor responders to ovarian stimulation during IVF/ICSI. Knowledge of the real-world use of the treatment is an essential complement to data from clinical trials and should aid decision making by clinicians, ultimately contributing to more successful IVF/ICI outcomes and thus improved fertility rates. Further studies with larger cohorts are warranted to confirm these observations and strengthen the evidence base for clinical decision-making in this specific group of patients.

## Data Availability

The original contributions presented in the study are included in the article/Supplementary Material, further inquiries can be directed to the corresponding author.
