# Supplementary material for: A multicentric real-world observational study to describe the use and efficacy of follitropin delta for IVF/ICSI procedures in patients at risk of hypo-response
Source: Front Reprod Health. 2025 Sep 10;7:1650946. doi: 10.3389/frph.2025.1650946 (PMC12457373; doi:10.3389/frph.2025.1650946)
Supplement: Supplementary file 1 [file Table1.docx]

**Supplementary Tables**

**Figures**

**Patients included in the overall population (EPS)**

N=47 (100%)

*POSEIDON 3 and 4 patients from the DELTA study*

**Patients excluded from the full analysis set (FAS)**

N=5 (10.6%)

**Non-eligible:**

- Patients without completed selection criteria (N=2)
- Patients without a start date of follitropin delta treatment (N=3)

**Patients included in the full analysis set (FAS)**

N=42 (89.4%)

**Patients excluded from the per-protocol population (PPS)**

N=7 (25.5%)

**Non-eligible:**

- Patients who did not complete the follitropin delta regimen (N=1)
- Patients with Follitropin alfa (N=6)

**Patients included in the per-protocol population (PPS)**

N=35 (74.5%)

Figure 1. Patient selection from DELTA for the post-hoc study.
